# Supplementary material for: Validation of a Classification Algorithm for Chronic Kidney Disease Based on Health Information Systems
Source: J Clin Med. 2022 May 11;11(10):2711. doi: 10.3390/jcm11102711 (PMC9144354; doi:10.3390/jcm11102711)
Supplement: Supplementary file 1 [file jcm-11-02711-s001.zip › jcm-1652320-supplementary.pdf]

Table S1. Staging of CKD according to KDIGO

		Stage
**eGFR value (mL/min/1.73 m^2)**		
	**≥90**	G1
	**60-89**	G2
	**45-59**	G3a
	**30-44**	G3b
	**15-29**	G4
	**<15**	G5
**Albuminuria value (mg/g creatinine)**		
	**<30**	A1
	**30-300**	A2
	**>300**	A3

Table S2. Diagnosis (code=ICD-9-CM), procedures (code=ICD-9-CM), outpatient services (code=Regional codification) and drugs (code=ATC), name and code. [6]

**Name**	**Code (diagnostic branch)**
Hospital Discharge Registry	
**Diagnosis**	
Diabetes with renal manifestations	250.4X
Hypertensive chronic kidney disease	403.XX
Hypertensive heart and chronic kidney disease	404.XX
Chronic glomerulonephritis	582.XX
Nephritis and nephropathy not specified as acute or chronic	583.XX
Chronic kidney disease (ckd)	585.XX
Renal failure, unspecified	586.XX
Renal sclerosis, unspecified	587.XX
Disorders resulting from impaired renal function	588.XX
Cystic kidney disease	753.1X
Chronic pyelonephritis	590.0X
Encounter for dialysis and dialysis catheter care	V56.X
Kidney replaced by transplant	V42.0
**Procedures**	
Hemodialysis	39.95
Peritoneal dialysis	54.98
Transplant of kidney	55.6X
Arteriovenostomy for renal dialysis	39.27
Creation of cutaneoperitoneal fistula	54.93
Revision of arteriovenous shunt for renal dialysis	39.42
Removal of arteriovenous shunt for renal dialysis	39.43
Venous catheterization for renal dialysis	38.95
Closed [percutaneous] [needle] biopsy of kidney	55.23
**Complex outpatient services for**	
Assess diagnosis of nephropathies	P583
Assess chronic kidney disease	P585A
Assess kidney transplant	P585B
Follow-up of kidney transplant patient	PV420
Outpatient Specialist Service Information System	
**Services**	
First ambulatory specialist visit (nephrology)	89.7 (29)
Ambulatory specialist visit (nephrology)	89.01 (29)
Measurement of urine albumin	90.33.4
Definition of the haemodialysis or peritoneal dialysis scheme (nephrology)	89.03 (29)

Hemodialysis or hemodiafiltration	39.95.X
Peritoneal dialysis	54.98.X
Venous catheterization for renal dialysis	38.95
Creation of cutaneoperitoneal fistula (peritoneal catheter)	54.93
Debriding of peritoneal catheter	39.99.1
Removal of peritoneal catheter	97.82
Revision of peritoneal catheter	97.29.1
Drug Dispensing Registry	
Erythropoietin	B03XA01
Darbepoetin alfa	B03XA02
Methoxy polyethylene glycol-epoetin beta	B03XA03
Polystyrene sulfonate	V03AE01
Sevelamer	V03AE02
Lanthanum carbonate	V03AE03
Sucroferric oxyhydroxide	V03AE05

Table S3: Characteristics of patients with CKD working standard (WS) and CKD algorithm (AL) and prevalence of CKD for both definitions; by sex and age class. Outpatient setting

	**CKD [WS]**					**CKD [AL]**				
	**N**	**%**	**prevalence %**	**95% CIs**		**N**	**%**	**prevalence %**	**95% CIs**	
**eGFR <60 mL/min/1.73 m²/ algorithm for identification of CKD**										
**Total**	**1,241**		**9.9**	**9.4**	**10.4**	**977**		**7.8**	**7.3**	**8.2**
**Sex**										
**Male**	650	52.4	13.8	12.8	14.8	573	58.6	12.1	11.2	13.1
**Female**	591	47.6	7.5	7.0	8.1	404	41.4	5.2	4.7	5.6
**Age class (years)**										
**19-44**	59	4.8	1.8	1.3	2.2	143	14.6	4.3	3.6	4.9
**45-64**	230	18.5	5.2	4.6	5.9	333	34.1	7.6	6.8	8.3
**65-74**	371	29.9	13.4	12.2	14.7	253	25.9	9.2	8.1	10.2
**75-84**	439	35.4	25.2	23.2	27.3	195	20.0	11.2	9.7	12.7
**85+**	142	11.4	45.5	40.0	51.0	53	5.4	17.0	12.8	21.1
**eGFR <30 mL/min/1.73 m²/ algorithm for identification of advanced CKD**										
**Total**	**202**		**1.6**	**1.4**	**1.8**	**463**		**3.7**	**3.4**	**4.0**
**Sex**										
**Male**	104	51.5	2.2	1.8	2.6	276	59.6	5.9	5.2	6.5
**Female**	98	48.5	1.3	1.0	1.5	187	40.4	2.4	2.1	2.7
**Age class (years)**										
**19-44**	15	7.4	0.5	0.2	0.7	94	20.3	2.8	2.3	3.4
**45-64**	54	26.7	1.2	0.9	1.6	205	44.3	4.7	4.0	5.3
**65-74**	51	25.2	1.9	1.4	2.3	99	21.4	3.6	2.9	4.3
**75-84**	59	29.2	3.4	2.5	4.2	50	10.8	2.9	2.1	3.7
**85+**	23	11.4	7.4	4.5	10.3	15	3.2	4.8	2.5	7.2

Table S4: Validity of CKD definitions on administrative data compared with the eGFR gold standard, by sex and age class. Outpatient setting

	**Sensi-tivity**	**95% CIs**		**Speci-ficity**	**95% CIs**		**PPV**	**95% CIs**		**NPV**	**95% CIs**	
**eGFR <60 mL/min/1.73 m²/ algorithm for identification of CKD**												
**Total**	**48.3**	**45.5**	**51.1**	**96.7**	**96.3**	**97.0**	**61.3**	**58.6**	**64.0**	**94.5**	**94.2**	**94.8**
**Sex**												
**Male**	54.9	51.1	58.8	94.7	94.0	95.4	62.3	58.8	65.8	92.9	92.4	93.5
**Female**	41.0	37.0	44.9	97.8	97.4	98.1	59.9	55.6	64.2	95.3	95.0	95.6
**Age class (years)**												
**19-44**	88.1	79.9	96.4	97.2	96.7	97.8	36.4	31.2	41.5	99.8	99.6	99.9
**45-64**	75.2	69.6	80.8	96.2	95.6	96.8	52.0	47.7	56.2	98.6	98.3	98.9
**65-74**	47.2	42.1	52.3	96.7	96.0	97.5	69.2	64.0	74.4	92.2	91.5	92.9
**75-84**	35.1	30.6	39.5	96.9	95.9	97.8	79.0	73.5	84.4	81.6	80.5	82.6

**85+**	31.7	24.0	39.3	95.3	92.1	98.5	84.9	75.7	94.1	62.6	59.7	65.4
**eGFR <30 mL/min/1.73 m^2 / algorithm for identification of advanced CKD**												
**Total**	**63.9**	**57.2**	**70.5**	**97.3**	**97.0**	**97.6**	**27.9**	**24.9**	**30.8**	**99.4**	**99.3**	**99.5**
**Sex**												
**Male**	68.3	59.3	77.2	95.6	95.0	96.2	25.7	22.2	29.3	99.3	99.1	99.5
**Female**	59.2	49.5	68.9	98.3	98.1	98.6	31.0	25.9	36.1	99.5	99.4	99.6
**Age class (years)**												
**19-44**	93.3	80.7	106.0	97.6	97.1	98.1	14.9	11.7	18.1	100.0	99.9	100.0
**45-64**	75.9	64.5	87.3	96.2	95.7	96.8	20.0	16.6	23.4	99.7	99.5	99.8
**65-74**	64.7	51.6	77.8	97.6	97.0	98.2	33.3	26.4	40.3	99.3	99.1	99.6
**75-84**	52.5	39.8	65.3	98.9	98.4	99.4	62.0	50.0	74.0	98.3	97.9	98.8
**85+**	43.5	23.2	63.7	98.3	96.8	99.8	66.7	44.8	88.6	95.6	94.1	97.1
